# Computer models to inform epilepsy surgery strategies: prediction of postoperative outcome

**DOI:** 10.1093/brain/awx067

**Published:** 2017-03-18

**Authors:** Marc Goodfellow, Christian Rummel, Eugenio Abela, Mark P. Richardson, Kaspar Schindler, John R. Terry

**Affiliations:** 1 College of Engineering, Mathematics and Physical Sciences, University of Exeter, Exeter, UK; 2 Centre for Biomedical Modelling and Analysis, University of Exeter, Exeter, UK; 3 EPSRC Centre for Predictive Modelling in Healthcare, University of Exeter, Exeter, UK; 4 Support Center for Advanced Neuroimaging (SCAN), University Institute for Diagnostic and Interventional Neuroradiology, University of Bern, Switzerland; 5 Institute of Psychiatry, Psychology and Neuroscience, King’s College London, London, UK; 6 Department of Neurology, University of Bern, Switzerland

Sir,

Recently, Sinha *et al.* (2017) published an article describing how a computer model interrogation of intracranial EEG recordings could be used to predict neurosurgical outcomes in people with medically intractable epilepsies. In this article, the authors derived functional networks from patient-specific intracranial EEG. They applied a dynamic model to these networks and used the output of the model to understand the relative ictogenicity of each node. This information was then used to predict which nodes should be removed to stop seizures from occurring, and these predictions were tested retrospectively on data from 16 patients with differing outcome after surgery. The authors demonstrated that this *in silico* approach could predict with 81.3% accuracy whether a patient would have good or bad outcome.

We would like to highlight that these results effectively replicate findings from a study we published in mid-2016 (Goodfellow *et al.* 2016). Therein we also extracted functional networks from patient-specific intracranial EEG and applied a dynamic model to these networks to understand the ictogenicity of each node. We also tested predictions from the model on 16 patients with varying outcome post-surgery and found that we could predict with 87.5% accuracy whether a patient would have good or bad outcome. In our article, we stated several benefits of this approach, including that it ‘allows alternative resection strategies to be tested *in silico*’, which Sinha *et al.* (2017) claim as one of the main novelties of their work, despite citing Goodfellow *et al.* (2016) as an example of ‘limited work in the context of epilepsy surgery’. In both studies, it was found that the optimal predicted resection would typically be smaller than the actual resection carried out.

Despite being broadly similar, there are technical differences in the two approaches (summarized in Table 1). Specifically, these relate to the choice of mathematical model that underpins the methodology, the selection of optimal resection strategies, the way in which the EEG functional network was constructed and the ‘ground truth’ data used to validate predictions. In Goodfellow *et al.* we used a neural mass model introduced by Wendling *et al.* (2002). Operating in the vicinity of a saddle-node on limit cycle bifurcation, this model approximates the transition to seizures in terms of increases in spiking dynamics. In contrast, Sinha *et al.* (2017) used a subcritical Hopf bifurcation, introduced in the context of epilepsy by Kalitzin *et al.* (2010). In both articles, intracranial EEG recordings were used to derive a patient-specific functional network; however, in Goodfellow *et al.* epochs during seizures were used, whereas in the Sinha *et al.* (2017) study, data from interictal epochs were used. Sinha *et al.* (2017) use network nodes displaying fastest transition into seizure dynamics as a proxy for ictogenic nodes, whereas in Goodfellow *et al.* we took a more mechanistic approach: nodes are deemed ictogenic if their removal from the network *in silico* reduces epileptiform dynamics.

**Table 1:** Comparison of key elements of the approaches of Goodfellow *et al.* and Sinha *et al.*

	Sinha *et al.*	Goodfellow *et al.*
Number of patients	16 (8 Engel I or II, 8 Engel III, IV or V)	16 (6 Engel I, 5 Engel II, 5 Engel IV)
Data type	Interictal epoch	Seizure epoch
Model	Subcritical Hopf bifurcation (Kalitzin *et al.*, 2010)	Neural mass model (Wendling *et al.*, 2002)
Accuracy	81.3%	87.5%
Sensitivity / specificity	87.5% / 75%	91% / 80%
Ground truth	Interpretation of descriptive accounts by experts and non-experts	Pre- and post-surgery image coregistration

Crucial to this type of study is obtaining the best possible approximation of the ‘ground truth’, i.e. the overlap between nodes in the computational model (located at intracranial EEG electrodes) and the regions of brain tissue resected. This allows predictions of the model to be validated. Ultimately the reported predictive capacity of both approaches is broadly similar in terms of sensitivity (91% versus 87.5%) and specificity (80% versus 75%) (Table 1). However, the approach used to determine the ‘ground truth’ is fundamentally different. In Goodfellow *et al.*, coregistration of pre- and post-resection images was used to objectively and quantitatively determine the overlap between resected brain tissue and nodes of the model. In contrast, Sinha *et al.* (2017) did not use imaging data, but instead estimated the anatomical extent of the resection qualitatively using descriptive accounts of the surgery that was performed. The estimation was performed by clinicians in four cases (the data from Massachusetts General Hospital) and by basic scientists in the other 12 cases (the publicly available data).

In summary, the replication of our earlier findings by Sinha *et al.* (2017) demonstrates robustness of *in silico* approaches to predict postsurgical outcome. A particularly important result is that predictions derived from interictal, rather than ictal data were found to be promising, which could be beneficial for patients undergoing presurgical monitoring, as seizures may not need to be observed. Such approaches offer exciting new possibilities to develop surgical and other treatment strategies for people with medically intractable epilepsies.

## Funding

M.G., M.P.R. and J.R.T. gratefully acknowledge the financial support of the EPSRC via grant EP/N014391/1. They further acknowledge funding from Epilepsy Research UK via grant number A1007 and the Medical Research Council via grant MR/K013998/1. The contribution of M.G. and J.R.T. was generously supported by a Wellcome Trust Institutional Strategic Support Award (WT105618MA). M.P.R. is supported by the National Institute for Health Research (NIHR) Biomedical Research Centre at the South London and Maudsley NHS Foundation Trust. C.R. and A.E. were supported by the Swiss National Science Foundation (grant SPUM 140332). K.S. is grateful for support from the Swiss National Science Foundation (grants 122010 and 155950).
